# Effect of a sodium fluoride rinse on the composition of early oral biofilms formed in situ on enamel and dentine

**DOI:** 10.1007/s00784-026-07121-1

**Published:** 2026-09-04

**Authors:** Luciana Pisenti Berto, Jingyu Ding, Letícia Oba Sakae, Samira Helena Niemeyer, Richard Johannes Wierichs, Antônio Pedro Ricomini-Filho, Hendrik Meyer-Lueckel, Tommy Baumann, Thiago Saads Carvalho

**Affiliations:** 1 Department of Restorative, Preventive and Pediatric Dentistry, School of Dental Medicine, University of Bern, Freiburgstrasse 7, Bern, 3010 Switzerland; 2 Department of Operative Dentistry and Periodontology, University of Bonn, Welschnonnenstrasse 17, Bonn, 53111 Germany; 3 Department of Biosciences, Faculdade de Odontologia de Piracicaba, Universidade Estadual de Campinas, Avenida Limeira 901, Piracicaba, São Paulo 13414-903 Brazil

**Keywords:** Fluoride, Microbiome, Sequencing, Enamel, Dentine, Caries

## Abstract

**Objectives:**

Dental caries results from dysbiotic shifts in the microbial community, so modulating biofilm composition represents a valid preventive strategy. The aim of this double-blind randomized crossover in situ study was to investigate how a fluoride rinse can modify the composition of the early oral biofilm formed on enamel and dentine.

**Materials and methods:**

Twelve volunteers wore mandibular appliances containing 3 enamel and 3 dentine specimens for 1 min, to allow basal pellicle formation, then rinsed (1 min) with a NaF solution (500 ppm F⁻) or deionized water (DW) and kept the appliance overnight (8 h). The composition of the biofilms was analysed using full-length 16 S rRNA gene sequencing.

**Results:**

Alpha and beta diversity did not differ between groups. Dominant genera were *Streptococcus* (61–71%), *Haemophilus* (15–24%), *Gemella* (4–6%), *Veillonella* (1–3%), and *Rothia* (1–2%), with no significant group differences. On both enamel and dentine, NaF rinse significantly increased the relative abundance of several *Streptococcus* spp., including *S. salivarius* and *S. toyakuensis*, while decreasing species of *Streptococcus*, *Neisseria*, and *Prevotella*. On enamel, NaF reduced *S. gordonii* and increased *S. oralis* and *S. parasanguinis*; on dentine, it increased species of *Rothia* and *Haemophilus*.

**Conclusions:**

NaF rinse induces subtle species-level changes in early biofilms formed on enamel and dentine, while overall community diversity and dominant genera remain stable.

**Clinical relevance:**

This exploratory study suggests that a 500 ppm NaF rinse is associated with subtle species-level shifts in early oral biofilms. Further studies are needed to determine whether these changes translate into functional benefits or improved oral health outcomes.

**Clinical trial registration:**

The study protocol was registered at ClinicalTrials.gov (NCT04033263).

**Supplementary Information:**

The online version contains supplementary material available at https://doi.org/10.1007/s00784-026-07121-1.

## Introduction

 Dental caries results from dysbiosis of the supragingival biofilm microbiota, driven by frequent sugar exposure and sustained acidic conditions [1]. Frequent sugar intake, coupled with reduced salivary flow rate and buffering capacity, further favours the dominance of acidogenic and aciduric microorganisms [2]. Therefore, changes in composition and metabolic activity of the biofilm can disrupt the ecological balance of the microbial community, promoting a cariogenic environment that drives mineral loss and caries lesion development [3].

Dental biofilm formation begins with bacterial adhesion to the acquired pellicle, a thin acellular film formed on the tooth surfaces from selectively adsorbed salivary components within minutes after tooth cleaning [4]. Modifications in pellicle composition or physicochemical properties may therefore affect initial microbial adhesion [5], potentially influencing the subsequent biofilm maturation. This process may differ on enamel and dentine, which vary in mineral content, surface energy, roughness, and organic matrix composition, leading to differences in pellicle constitution and microbial colonization [6, 7]. With the aim of maintaining microbial homeostasis and preventing caries development, various approaches have been investigated to modulate oral biofilm composition and metabolism, including antimicrobial agents, probiotics, prebiotics, arginine supplementation, and compounds that interfere with bacterial adhesion [8]. However, the influence of fluoride in the biofilm composition remains poorly characterized.

Fluoride is the most used substance in caries control due to its effects on de- and remineralization [9]. It is also known that fluoride has inhibitory effects on the metabolism of cariogenic streptococci [10], impairing the activity of key enzymes such as enolase and F-ATPase and reducing the secretion of glucosyltransferases [11–13]. In addition, fluoride may also influence early biofilm formation through interactions with the pellicle and bacterial adhesion processes [5]. Recent studies indicate that fluoride-containing rinses can reduce bacterial attachment to pellicle-covered enamel surfaces and may alter pellicle composition [5, 14], although the magnitude of these effects on the microbial composition of the dental biofilm remain unknown.

Understanding how a fluoride rinse influences microbial colonization and early biofilm composition could provide insight into additional mechanisms underlying its cariostatic effects. In addition, differences in the microbial composition of early biofilms formed on enamel and dentine in the presence of fluoride remain poorly characterized. Therefore, this study investigated the effect of a sodium fluoride rinse on the composition of early biofilms formed on enamel and dentine.

## Materials and methods

### Experimental design

This was a double-blind, randomised, two-phase crossover study. Twelve volunteers wore customised intraoral appliances containing enamel and dentine specimens. Participants wore the appliance during the intervention (rinsing with a solution) and for eight hours overnight to allow biofilm formation. To minimise carryover effects, 10-day led-in and washout periods were implemented before the first and between the two phases. The evaluated factors were:

- Mouth rinse solutions:
  - Negative control: deionized water (DW);
  - Test solution: 500-ppm F^-^ sodium fluoride solution, pH 5.8 (NaF)
- Substrate:
  - Enamel;
  - Dentine

### Ethical approval and study registration

The study was conducted in accordance with the Declaration of Helsinki and approved by the local ethics committee (KEK - Project ID 2022 − 01904). Study protocol was registered on ClinicalTrials.gov (NCT04033263). Informed consent was obtained from subjects before the study commencement. Extracted human teeth were collected and pooled in the dental clinics of the university for the preparation of specimens. The KEK considers pooled samples to be irreversibly anonymized and therefore, no prior ethical approval for this procedure was required.

### Study population and inclusion/exclusion criteria

Twelve adult participants from both genders were recruited and informed about the study. After agreeing to take part, they read and signed an informed consent statement. Inclusion criteria were good general health and age ≥ 18. Exclusion criteria were pregnancy, smoking, use of antibiotics in the last three months, and presence of active caries lesions, gingival bleeding or periodontal disease, verified during anamnesis and clinical examination.

### Randomization and blinding

All participants were identified by a unique study number. They were randomly assigned to the treatment solutions according to a computer-generated list. Information on the treatment allocation was kept secret by an investigator not involved in the interventions or data analysis until the statistical analyses were completed. Participants and outcome assessor were blinded to group allocation, as the solutions were identical in appearance and labelling, and no differences in taste were reported by any participant.

### Specimen preparation

A total of 144 enamel and dentine specimens were prepared respectively from the crown and roots of human molars. Teeth were firstly cleaned with periodontal curettes and kept in 2% chloramine solution, at 4 °C, until specimen preparation. Cylindric specimens measuring 3 mm diameter x 1 mm thickness were drilled from the buccal sides of the crowns or roots, with a trephine bur (Zepf, Germany) attached to a drilling machine (Proxxon Fine Bore Grinder, Luxemburg), under water cooling. Pulp surfaces of specimens were flattened, while buccal surfaces were ground and polished with decreasing-grit silicon carbide papers (15 μm to 5 μm - Struers, Denmark), under constant water cooling (TegraPol-15, Struers, Denmark) [15]. After the polishing procedures, specimens were inspected to exclude those presenting cracks or structural defects, and the smear layer was removed from the surfaces using 3% sodium hypochlorite in ultrasonic bath (10 min), followed by rinsing with DW. Specimens were then disinfected in 70% ethanol in ultrasonic bath (30 min) and rehydrated in sterile DW (30 min) [16]. Finally, they were stored in sterile water at 4 °C until use.

### Intraoral appliances

Bilateral mandibular intraoral appliances were fabricated from acrylic resin (Paladur, Heraeus Kulzer GmbH, Germany) using individual plaster models obtained from lower-jaw impressions [17]. Three niches (4 × 4 × 2 mm) were created on each buccal side (premolar and molar regions) with a spherical diamond bur. Three enamel (left side) and three dentine (right side) specimens were fixed within the niches using a thin wax layer, leaving a 1-mm gap from the acrylic surface to prevent friction with the buccal mucosa.

### Interventions

Before the study, participants were trained in all procedures and received written instructions. They were provided with a toothpaste (1450 ppm F⁻ as NaF; Colgate Fresh Gel, Colgate-Palmolive, Switzerland) and a standard toothbrush (Pro-interdental, Trisa AG, Switzerland) for use during the 10-day lead-in phase and throughout the study. No additional oral hygiene products were permitted, and participants were asked to maintain their usual brushing frequency and technique. For the experimental procedure, participants brushed their teeth 30 min before bedtime. Thirty minutes after brushing, they inserted the intraoral appliance, allowed pellicle formation for 1 min, and rinsed for 1 min with 15 mL of the assigned solution. The appliance was then worn overnight for 8 h. In the morning, it was removed and placed in a container with sterile moist gauze before being returned to the investigators. Following a 10-day washout period, the second intervention was performed using the same protocol.

### Biofilm harvesting and DNA extraction

Biofilm-covered specimens were removed from the appliances with a sterile spatula and transferred individually to microcentrifuge tubes containing phosphate buffer solution (pH 7.2). Following vigorous mixing, samples were sonicated for 1 min to facilitate biofilm detachment. Specimens were then discarded, and the biofilm suspensions were stored at −80 °C until analysis. Prior to DNA extraction, samples were pooled by replicate. Each pool was generated by combining equal volumes of the corresponding replicates (replicate 1, 2, or 3) from all 12 participants. Consequently, each treatment/substrate group was represented by three pooled biological samples (*n* = 3). Pooling was performed to increase the biomass for DNA extraction. DNA was extracted using the ZymoBIOMICS DNA Miniprep Kit (Zymo Research, USA) according to the manufacturer’s instructions. Mechanical lysis was performed with a FastPrep-96 system (MP Biomedicals, USA) for five cycles of 60 s each at 1800 rpm and 1-min ice-cooling intervals.

### Library preparation and sequencing

Genomic DNA concentration was measured using a Qubit 4.0 fluorometer (Thermo Fisher Scientific, USA). Integrity and purity were assessed by capillary electrophoresis (FEMTO Pulse, Agilent, USA) and spectrophotometry (DS-11, DeNovix, USA), respectively. Full-length bacterial 16 S rRNA genes (V1–V9) were amplified using barcoded primers targeting positions 27 F (AGRGTTYGATYMTGGCTCAG) and 1492R (RGYTACCTTGTTACGACTT), each primer containing a 10-nt sample-specific barcode and PacBio adapter sequences. Libraries were prepared according to the PacBio Kinnex 16 S rRNA amplicon protocol. Three positive controls (ZymoBIOMICS Microbial Community DNA Standards I and II, and ATCC MSA-3001) and two negative controls (elution buffer and PCR master mix without DNA template) were included. Amplicons were quantified using the Qubit dsDNA High Sensitivity Assay and their size distribution verified by capillary electrophoresis. Final Kinnex libraries (~ 18 kb) were assessed for concentration, size distribution, and purity. SMRTbell libraries were generated using the PacBio SMRT Link v13.1 workflow. Sequencing primer annealing, polymerase binding, and complex purification were performed according to the manufacturer’s instructions (Revio polymerase kit). A sequencing control DNA was spiked into the final complex prior to loading. Libraries were sequenced on a PacBio Revio platform using 25 M SMRT Cells with adaptive loading at an on-plate concentration of 160 pM and a 30-hour movie time.

### Bioinformatics and statistical analyses

Data analysis was performed before treatment unblinding. The analytical unit for all microbiome analyses was the pooled sample (*n* = 3 per treatment/substrate condition). Therefore, statistical inference was restricted to the treatment/substrate level, and the microbiome analyses should be considered exploratory due to the small number of biological replicates and the inability to perform participant-level paired analyses despite the crossover study design. Raw PacBio HiFi reads were processed using QIIME2 (version 2024.5). Reads were demultiplexed, quality-filtered, and processed with DADA2 for denoising and chimera removal, generating high-resolution amplicon sequence variants (ASVs). Taxonomic assignment of ASVs was conducted using a naive Bayes classifier trained on the SILVA SSU 138.2 database. Alpha diversity was evaluated using the Shannon diversity index and compared with the Mann-Whitney test. Beta diversity was evaluated using Bray-Curtis dissimilarity for Amplicon Sequence Variant (ASV) and tested using PERMANOVA with 999 permutations. Exploratory differential abundance analyses at the ASV and genus levels were performed using DESeq2 in R (version 1.50.2). The ASV count table generated after DADA2 denoising and chimera removal was filtered using the default parameters of the HiFi-16 S Nextflow workflow, retaining ASVs with a minimum total abundance of five reads across the dataset and present in at least one sample, before being used as input for DESeq2. Normalization and dispersion parameters were estimated using the standard DESeq2 workflow. In all comparisons, *p*-values were adjusted for multiple testing using the Benjamini–Hochberg correction. Level of significance was set at 5%. Log-ratios from relative abundances of *Prevotella* spp. to *Haemophilus*, *Neisseria* and *Rothia* spp were calculated to analyze the potential of the biofilms for causing caries [18].

## Results

**Fig. 1:**
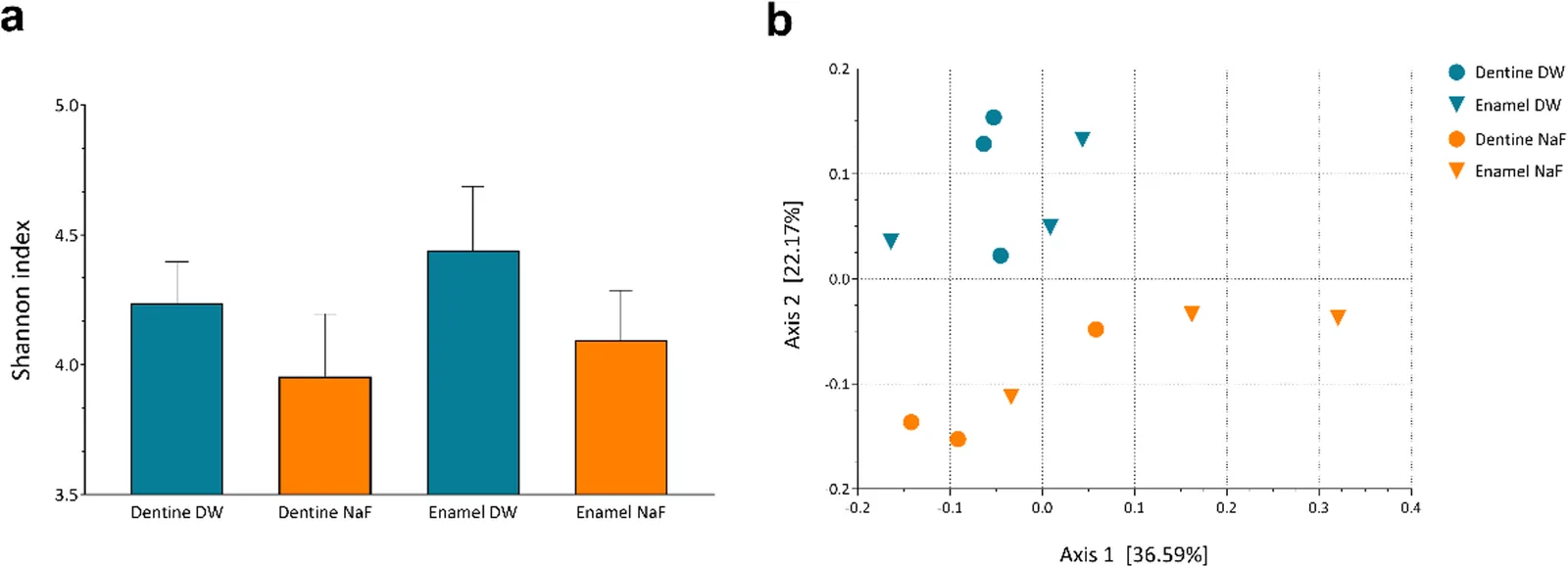
Alpha and beta diversity analyses biofilms formed in situ on enamel and dentine following rinsing with deionized water (DW) or a 500-ppm F^−^ sodium fluoride solution (NaF). Analyses were performed on pooled biofilms samples (*n* = 3), each sample representing one pool from 12 participants. (**a**) Alpha diversity assessed using the Shannon index, presented as the median and interquartile range. (**b**) Beta diversity assessed by principal coordinates analysis (PCoA) based on Bray-Curtis dissimilarity for ASV values. Data points correspond to the pooled replicate samples per group (*n* = 3). No statistically significant differences were observed between groups for either metric (*p* > 0.05)

All participants completed the study, and no adverse events were reported. The twelve participants (5 males and 7 females) aged from 22 to 46 years (age average ± SD = 32.7 ± 8.1 years). Because the analyses were performed on pooled samples, the results represent exploratory treatment-level observations and should not be interpreted as participant-level responses. An average of 212,361 sequence reads were obtained per sample, each with a length of ~ 1500 bp. Species-level classification was possible for 82.3% of reads and genus-level classification for 17.0%, with 0.7% remaining unassigned. ASVs clustering at a similarity threshold of 97% or higher identified 228 taxa at species level, distributed across 58 genera. Shannon index medians ranged from 3.83 to 4.68 and did not differ significantly between the DW and NaF groups for either enamel or dentine biofilms, suggesting no consistent effects of fluoride on community richness and evenness (*p* > 0.05). Similarly, Bray-Curtis dissimilarity revealed no significant differences in beta diversity between groups (*p* > 0.05). A non-significant trend toward lower Shannon index values and altered community composition was observed in NaF-treated biofilms on both substrates (Fig. 1).

**Fig. 2:**
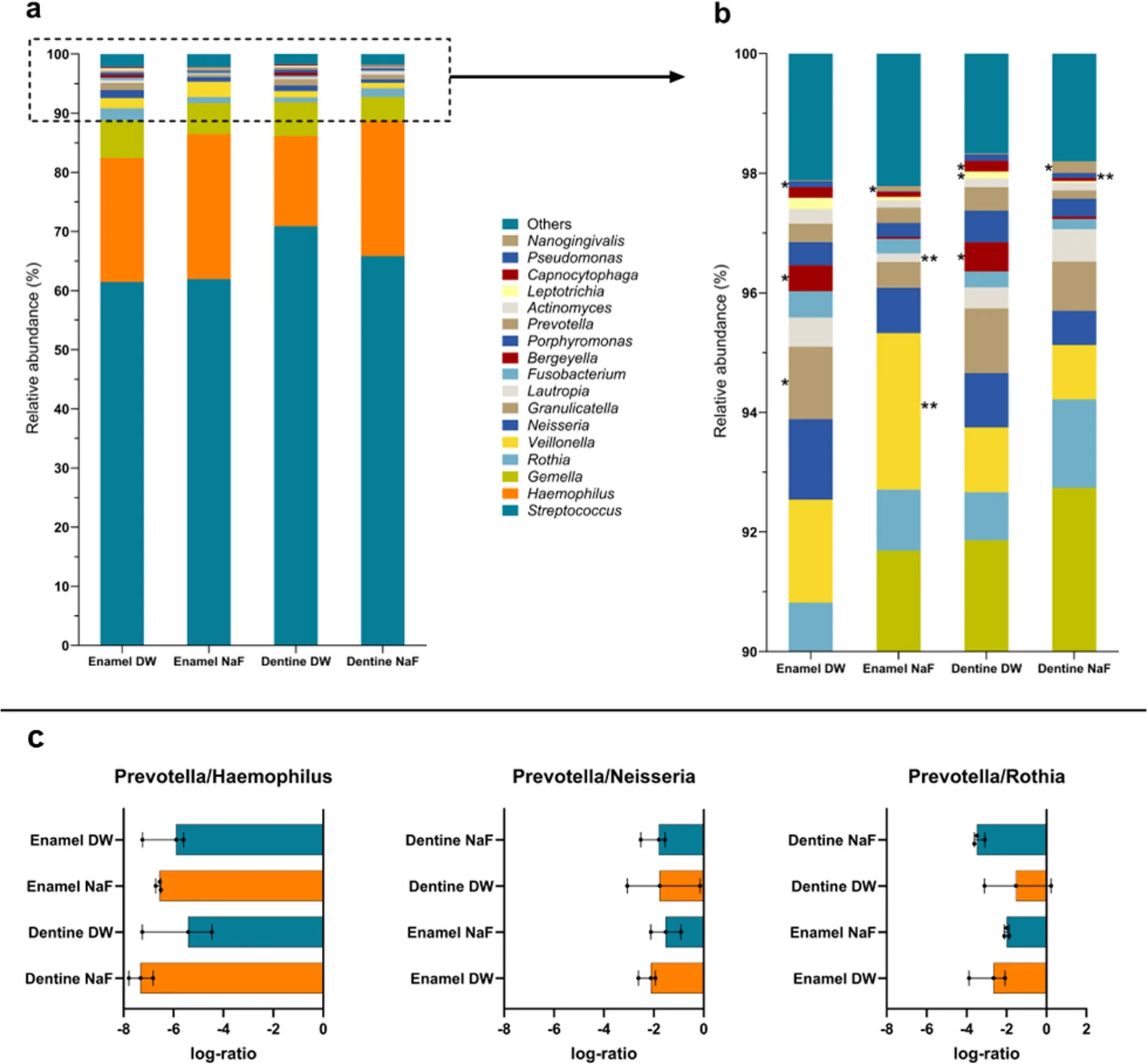
Genera identified in the biofilms formed on enamel and dentine after rinsing with deionised water (DW) or a 500-ppm F^−^ sodium fluoride solution (NaF). Analyses were performed on pooled biofilms samples (*n* = 3), each sample representing one pool from 12 participants. (**a**) Relative abundance of genera. (**b**) Least abundant genera (< 5%). * Indicates a significant difference between treatments within the same substrate (*p* < 0.05). ** Indicates a significant difference between substrates within the same treatment (*p* < 0.05). (**c**) Log-ratios of relative abundances of *Prevotella* spp. to *Haemophilus*, *Neisseria* and *Rothia* spp. Ratios did not differ significantly between groups (*p* > 0.05)

The samples harboured a typical oral microbiome profile, with Bacillota (formerly Firmicutes) as the dominant phylum in all groups, and abundances averaged at 70.7% and 78.8% for DW, and 70.2% and 71.6% for NaF, in enamel and dentine, respectively. On enamel, *Streptococcus* was the dominant genus, followed by *Haemophilus*, *Gemella*, *Rothia* and *Veillonella*, with average abundances of 61.4/61.9%, 21.0/24.5%, 6.4/5.2%, 2.0/1.0% and 1.7/2.6% for DW/NaF, respectively. A similar pattern was observed for the biofilms formed on dentine, with average abundances of 70.9/65.8% (Streptococcus), 15.2/22.9% (*Haemophilus*), 5.7/4.0% (*Gemella*), 1.1/0.9% (*Veilonella*) and 1.1/0.8% (*Granulicatella*) for DW/NaF, respectively (Fig. 2a).

At the genus level, significant differences were observed for low abundant groups, as shown in Fig. 2b. On enamel, NaF decreased *Granulicatella* (log_2_FC = −1.09, *p* = 0.03), *Bergeyella* (log_2_FC = −3.26, *p* < 0.01) and *Pseudomonas* (log_2_FC = −2.53, *p* = 0.03), and increased *Nanogingivalis* (log_2_FC = 3.13, *p* < 0.01), compared to DW. On dentine, NaF decreased *Bergeyella* (log_2_FC = −3.47, *p* < 0.01), *Leptotrichia* (log_2_FC = −2.02, *p* = 0.03) and *Capnocytophaga* (log_2_FC = −1.65, *p* = 0.03) and increased *Nanogingivalis* (log_2_FC = 4.05, *p* < 0.01), compared to DW. After rinsing with NaF, biofilms formed on enamel showed higher levels of *Veillonella* (log_2_FC = 1.45, *p* = 0.04), and lower levels of *Lautropia* (log_2_FC = −2.03, *p* = 0.04) and *Pseudomonas* (log_2_FC = −2.57, *p* = 0.04) compared to biofilms formed on dentine. No significant differences were observed in the abundance of genera when comparing the biofilms formed on enamel and dentine after rinsing with DW (*p* > 0.05). Comparisons of the relative abundance log-ratios of *Prevotella* spp. to *Haemophilus*, *Neisseria* and *Rothia* spp showed no significant differences between the groups (*p* > 0.05 - Fig. 2c).

**Fig. 3:**
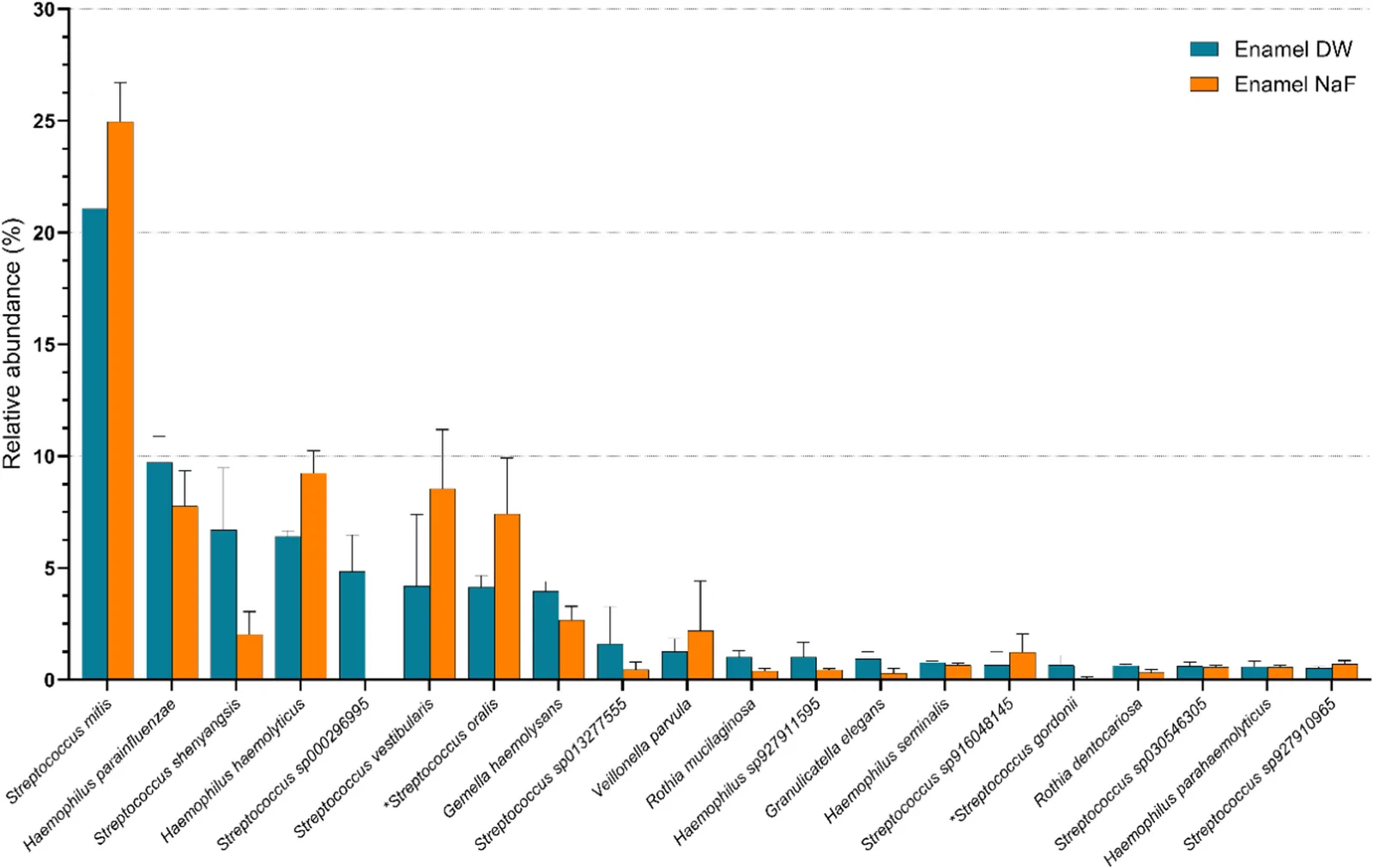
The 20 most abundant species observed in the pooled biofilm samples formed on enamel after rinsing with deionised water (DW) or a 500-ppm F^−^ sodium fluoride solution (NaF). Each sample (*n* = 3) represented one pool from 12 participants. Relative abundances are represented by mean and standard deviations. * indicates *p* < 0.05

**Fig. 4:**
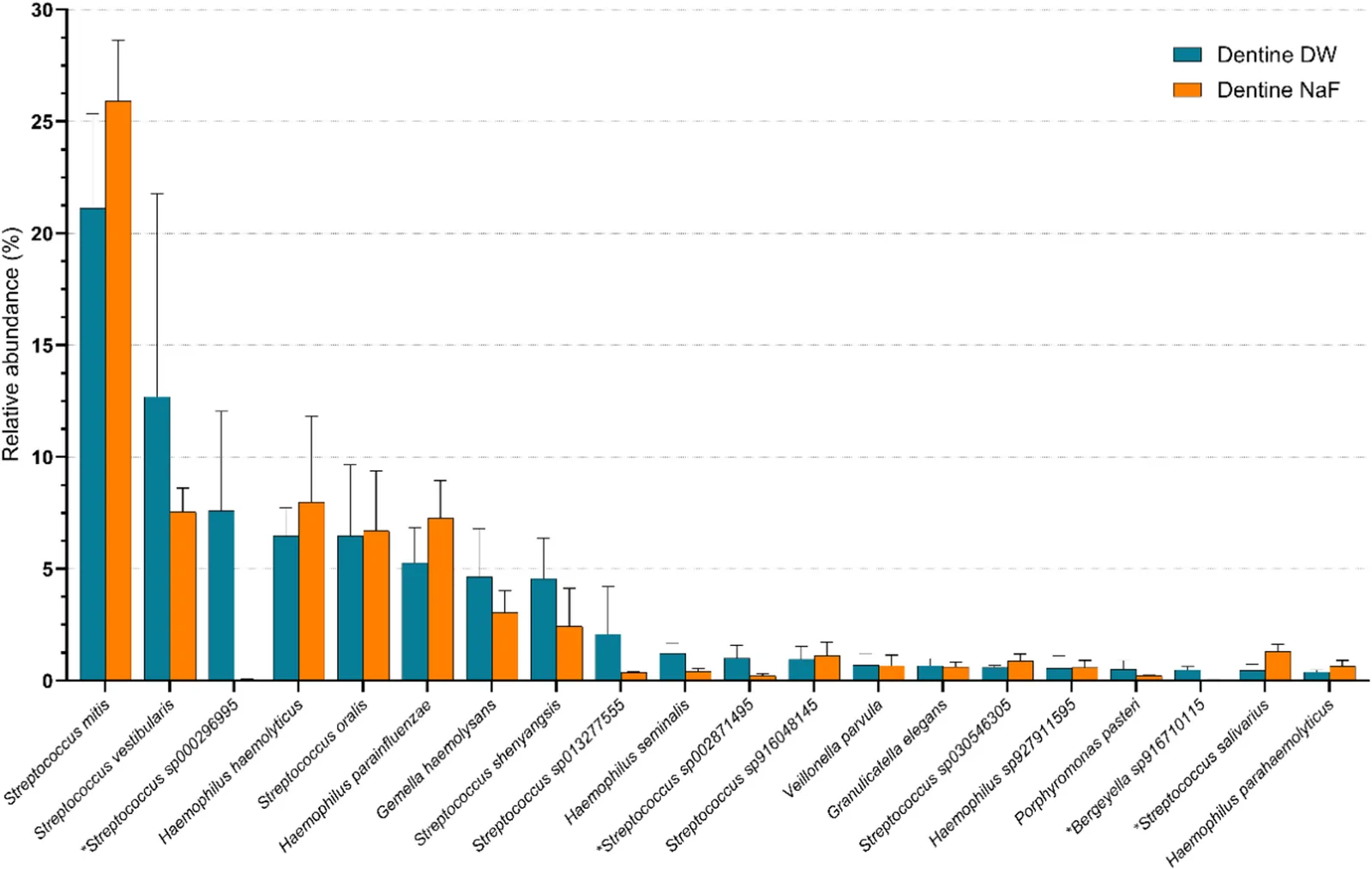
The 20 most abundant species identified in the pooled biofilm samples formed on dentine after rinsing with deionised water (DW) or a 500-ppm F^−^ sodium fluoride solution (NaF). Each sample (*n* = 3) represented one pool from 12 participants. Relative abundances are represented by mean and standard deviations. * indicates *p* < 0.05

*S. mitis* was the most abundant species across all groups (mean relative abundance: 23.3%). The most abundant species detected in enamel and dentine biofilms are shown in Figs. 3 and 4, respectively.

NaF significantly altered the abundance of multiple species on both substrates (Table 1), including reductions in *Prevotella aurantiaca*, *Neisseria elongata*, and uncultivated *Streptococcus* and *Bergeyella* species, and increases in *S. symci*, *S. toyakuensis*, *S. salivarius*, and *Neisseria mucosa*. Several additional changes were substrate-specific, with enamel biofilms showing shifts mainly within *Streptococcus* species and dentine biofilms exhibiting broader changes involving *Neisseria*, *Prevotella*, *Capnocytophaga*, *Rothia*, and *Haemophilus* taxa. Notably, many differentially abundant species were present at low relative abundances (< 0.1% - Table 1).

**Table 1:** List of species with significant changes in relative abundance (RA) identified in biofilms formed after a rinse with a 500-ppm fluoride solution (NaF) compared to a rinse with distilled water. Species are organized by decreasing log_2_FC values. Each sample (*n* = 3) represented one pool from 12 participants

**Species**		Log_2_FC (p-value)	**RA after NaF** **(%, mean ± SD)**
**Enamel biofilm**			
Reduced species			
	*Streptococcus *sp000296995	-6.74 (p <0.001)	0.031 ± 0.015
	*Prevotella aurantiaca*	-5.62 (p <0.001)	<0.001 ± <0.001
	*Streptococcus *sp903645285	-5.31 (p <0.001)	0.003 ± 0.002
	*Streptococcus *sp001808665	-3.46 (p = 0.022)	0.003 ± 0.003
	*Bergeyella *sp916710115	-3.18 (p <0.001)	0.033 ± 0.019
	*Streptococcus rubneri*	-3.11 (p <0.001)	0.017 ± 0.007
	*Neisseria elongata*	-3.04 (p <0.001)	0.009 ± 0.001
	*Streptococcus gordonii*	-2.75 (p = 0.012)	0.068 ± 0.072
	*Streptococcus *sp905221035	-2.66 (p <0.001)	0.707 ± 0.151
	*Streptococcus *sp001553685	-1.78 (p = 0.022)	0.025 ± 0.007
Increased species			
	*Streptococcus toyakuensis*	8.34 (p = 0.003)	0.020 ± 0.024
	*Nanogingivalis* sp015257795	4.82 (p = 0.002)	0.053 ± 0.065
	*Streptococcus anginosus*	4.64 (p = 0.019)	0.022 ± 0.028
	*Streptococcus salivarius*	3.98 (p <0.001)	2.748 ± 0.674
	*Streptococcus parasanguinis*	3.22 (p = 0.001)	0.327 ± 0.360
	*Streptococcus symci*	3.02 (p <0.001)	0.102 ± 0.050
	*Streptococcus* sp902363395	2.84 (p = 0.029)	0.089 ± 0.040
	*Prevotella pallens*	2.75 (p = 0.013)	0.017 ± 0.006
	*Centipeda noxia*	2.63 (p = 0.020)	0.021 ± 0.005
	*Neisseria mucosa*	2.11 (p = 0.041)	0.120 ± 0.110
	*Streptococcus oralis*	1.37 (p = 0.040)	7.421 ± 2.498
**Dentine biofilm**			
Reduced species			
	Unassigned taxa of *Rothia*	-7.50 (p <0.001)	Not present
	*Streptococcus* sp027665725	-6.83 (p = 0.010)	Not present
	*Streptococcus *sp000296995	-6.65 (p <0.001)	0.031 ± 0.015
	*Prevotella salivae*	-6.59 (p = 0.027)	Not present
	*Prevotella aurantiaca*	-5.22 (p = 0.041)	Not present
	*Veillonella tobetsuensis*	-3.53 (p = 0.027)	0.003 ± 0.003
	*Neisseria flava*	-3.52 (p = 0.002)	0.006 ± 0.008
	*Bergeyella *sp916710115	-3.29 (p <0.001)	0.038 ± 0.010
	*Neisseria* sp000186165	-2.85 (p = 0.003)	0.020 ± 0.019
	*Streptococcus* sp905221035	-2.76 (p <0.001)	0.037 ± 0.013
	*Capnocytophaga gingivalis*	-2.61 (p = 0.003)	0.008 ± 0.003
	*Leptotrichia hongkongensis*	-2.48 (p = 0.041)	Not present
	*Prevotella nanceiensis*	-2.10 (p = 0.005)	0.019 ± 0.003
	*Streptococcus* sp002871495	-1.92 (p = 0.034)	0.219 ± 0.087
	*Neisseria elongata*	-1.75 (p = 0.037)	0.012 ± 0.002
	*Capnocytophaga sputigena*	-1.71 (p = 0.042)	0.022 ± 0.009
Increased species			
	*Rothia* sp001836735	8.94 (p <0.001)	0.036 ± 0.015
	Unassigned viridans streptococcus	7.07 (p = 0.003)	0.010 ± 0.009
	*Streptococcus toyakuensis*	6.57 (p = 0.027)	0.008 ± 0.011
	*F0058* sp000163695	5.68 (p = 0.008)	0.003 ± 0.001
	*Neisseria sicca*	4.96 (p = 0.008)	0.024 ± 0.014
	*Nanogingivalis* sp916438495	4.80 (p <0.001)	0.173 ± 0.127
	*Streptococcus* sp002238115	4.48 (p <0.001)	1.665 ± 1.119
	*Haemophilus* sp030405845	4.39 (p = 0.041)	0.008 ± 0.007
	*Streptococcus symci*	4.15 (p <0.001)	0.143 ± 0.075
	Unassigned taxa of *Haemophilus*	3.17 (p <0.001)	5.582 ± 4.748
	*Streptococcus* sp001808665	3.11 (p = 0.027)	0.203 ± 0.150
	*Rothia aeria*	2.12 (p = 0.038)	0.304 ± 0.208
	*Neisseria mucosa*	2.08 (p = 0.034)	0.052 ± 0.022
	*Streptococcus salivarius*	1.92 (p <0.001)	1.312 ± 0.308
	*JABCPE02* sp013333255	1.16 (p = 0.041)	0.185 ± 0.057

**Fig. 5:**
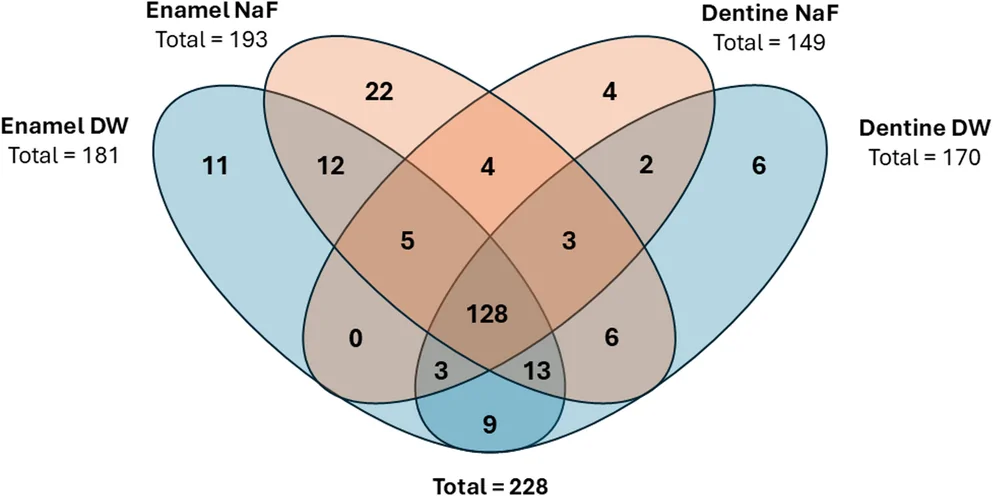
Venn diagram showing the number of species detected in each group (*n* = 3). Analyses are considered exploratory due to the pooling of samples. Each sample represented one pool from 12 participants

After NaF rinse, several species were observed exclusively on enamel (22 taxa) or dentine (4 taxa), as shown in Fig. 5. These observations are descriptive, as the detection of low-abundance taxa is influenced by sequencing depth, filtering thresholds, and the pooling strategy used in this study. Comparisons between the two substrates within the same treatment identified a small number of differentially abundant taxa (for each treatment, four taxa differed between substrates); however, all occurred at very low relative abundances and were considered biologically irrelevant. The complete list of relative abundances for the identified genera and species is provided in the Supplementary material available online.

## Discussion

Our study is the first to evaluate the influence of a fluoride rinse on the composition of early biofilms formed in situ on both enamel and dentine surfaces. The analysis of the oral bacterial profile suggests that fluoride exposure does not significantly influence richness, diversity, or the relative abundance of dominant genera in the biofilms, but it may induce significant changes in the relative abundances of less present genera and several species, in both substrates. In addition, we observed that fluoride may affect differently the colonization on enamel and dentine. It is important to highlight that our findings reflect exploratory observations of the microbiome analysis using pooled samples, so participant-level interpretation is not possible.

In our study, biofilms were formed after toothbrushing, minimizing dietary nutrient availability in the oral cavity. In addition, caries-free participants with no periodontal disease were selected. This design mitigated the influence of dietary factors or unbalanced oral microbial communities in the analysis, thereby isolating the effect of fluoride on the biofilm composition. As a result, we observed low log-ratios of the relative abundance of *Prevotella* to *Haemophilus*, *Neisseria* and *Rothia*, suggesting absence of disease [18]. However, this participant selection limits the generalizability of the findings to individuals at higher risk of caries, who may benefit most from fluoride exposure. Future studies could assess the effects of fluoride in caries-active individuals, dysbiotic microbial communities or in the presence of fermentable carbohydrates.

Previous in vitro studies investigated the impact of sodium fluoride (NaF) on oral biofilm composition. Gondo et al. [19] reported that continuous exposure to NaF (1450 ppm F^−^) for three days did not significantly affect microbial richness or diversity in saliva-derived polymicrobial biofilms grown in hydroxyapatite discs, although it reduced the abundance of specific taxa such as *Bulleidia* spp. Additionally, the authors observed a decrease in microbial viability of the biofilms associated with NaF treatment. Similarly, Vale et al. [12] demonstrated that a 3-minute exposure to high concentrations of fluoride (5000 ppm as NaF), twice a day for four days, reduced viable counts of *Streptococcus mutans*, *Streptococcus gordonii*, *Veillonella parvula*, and *Prevotella nigrensis* in multispecies biofilms formed on dentine specimens in vitro. In both studies, biofilms were initially grown for over 24 h and later exposed to continuous or repeated fluoride treatments. Changes in microbial composition and viability were associated with the inhibitory activity of fluoride against the established biofilms. Similar effects were observed in our clinical setting, with reduced relative abundance of *S. gordonii* and *Prevotella* species (*P. aurantiaca*) on enamel, and *Veillonella* species on dentine (*V. tobetsuensis)* after a single application of a 500-ppm fluoride NaF solution only prior to biofilm formation. Our findings therefore likely reflect interactions between fluoride and the acquired pellicle that may modulate early microbial adhesion in addition to directly inhibiting bacteria.

Our findings may be explained by previous in vitro evidence suggesting that fluoride modulates biofilm composition through multiple mechanisms [5, 14]: (i) altering the composition and physicochemical properties of the acquired pellicle, thereby affecting bacterial adhesion and early colonization [5, 14, 20], and (ii) inhibiting specific oral taxa [10, 12].

The modification of the salivary pellicle has been investigated as a possible strategy for caries prevention by engineering the biofilm formation [21]. Fluoride can modify the pellicle structure and composition [20] by interacting with salivary proteins and altering their adsorption patterns onto the tooth surface, though the exact mechanism is still not clear [14]. For instance, negatively charged salivary proteins such as statherin, histatin 1, acidic proline-rich protein 1 (acidic PRP-1) and calmodulin decreased their adsorption onto hydroxyapatite treated with high concentrations of fluoride [20]. These proteins are mostly related to the basal layer of the pellicle. Moreover, in their study [20], the authors applied fluoride to the hydroxyapatite disc before exposure to saliva, whereas in our study, we first formed clinically the basal layer (1 min), so we do not expect a decrease in statherin, histatin 1, acidic PRP-1 in our basal pellicles, although this could be altered in the modulation stages. Interestingly, acidic PRP-1 enhances the adhesion of the cariogenic *S. mutans* and *Actinomyces viscosus* to tooth surfaces [22, 23], while calmodulin mediates binding to *Candida albicans*, associated with early childhood caries [24] and a key fungal pathogen in oral candidiasis [25]. In addition, some proteins from the pellicle may bind fluoride, altering its physicochemical properties [5]. Thus, fluoride may influence the establishment of a less pathogenic biofilm by affecting species-specific interactions with the pellicle and reducing binding sites for bacterial adhesins [14]. These changes can explain the observed reduction in the abundance of specific taxa.

Additionally, fluoride inhibits oral microorganisms in vitro. It can diffuse into bacterial cells as hydrogen fluoride (HF), particularly under acidic conditions, working as a transmembrane proton transporter. Inside the bacterial cell, it dissociates and leads to intracellular acidification and metabolic disruption through the inhibition of essential bacterial enzymes [10]. For instance, fluoride inhibits enolase, a key glycolytic enzyme, at low concentrations such as 10 ppm [12, 26], leading to reduced ATP generation and acid production. So, the 500-ppm fluoride rinse used in present study may have reached the sufficient intracellular concentrations to inhibit enolase in some species. In addition, fluoride interferes with proton-translocating ATPases, impairing the ability of bacteria to regulate intracellular pH and maintain energy balance, particularly in the presence of aluminium [11]. Also, fluoride does not reduce the activity of glucosyltransferases, but their secretion, thereby impairing the synthesis of water-insoluble extracellular polysaccharides in *S. mutans* [13, 27]. These effects are particularly relevant for cariogenic *Streptococcus* species, whose pathogenicity depends on acid production, acid tolerance, and biofilm formation. Consistent with this, NaF rinsing was associated with a reduced relative abundance of several *Streptococcus* species, whereas others were enriched, suggesting differences in susceptibility to fluoride among species [28, 29]. Despite the inhibitory effects attributed to fluoride in vitro, its impact on bacteria under clinical conditions appears limited, with the anti-cariogenic effect in therapeutic concentrations being largely related to limiting demineralization and enhancing remineralization of dental hard tissues [30].

Levels of *S. salivarius*, *S. symci* and *S. toyakuensis* increased in both enamel and dentine following NaF rinsing. *S. salivarius* is a commensal species frequently found in health-associated oral communities [31, 32]. It contributes to colonization resistance by inhibiting the growth of oral pathogens through both direct antagonism and competitive adhesion [33]. Strains of this species have also been explored for their potential as probiotics. For instance, *S. salivarius* K12 has shown promising results in the management of halitosis, due to its production of the lantibiotics salivaricin A2 and salivaricin B, which reduce volatile sulfur compound-producing bacteria [32]. Another strain, *S. salivarius* M18, produces bacteriocins that inhibit *S. mutans*, as well as enzymes such as dextranase and urease, which contribute to reducing biofilm formation and acidification [34]. Therefore, the enrichment of *S. salivarius* observed in the present study may be beneficial for maintaining the ecological balance of the oral microbiome and potentially reducing caries progression. This finding is particularly relevant because *S. salivarius* remained one of the most abundant species following the fluoride rinse, accounting for 2.75% and 1.31% of the microbiome on enamel and dentine, respectively. Although several other species also showed statistically significant changes in relative abundance, most were detected at low abundances (lower than 0.1%), so their contribution to the overall community may be limited. *S. symci* and *S. toyakuensis* are recently characterized species of the mitis group [35, 36]. While the role of *S. symci* in the oral microbiome remains unclear, *S. toyakuensis* has attracted attention due to its resistance to multiple antibiotic classes, acting as a potential reservoir of multidrug-resistance genes [36, 37]. However, this characteristic is not unique to this species, as several oral streptococci can act as reservoirs of resistance genes and contribute to their dissemination through horizontal gene transfer [38]. Therefore, the observed increase in the relative abundance of *S. toyakuensis* following NaF rinsing and its potential implications for antimicrobial resistance warrant further investigation.

In our study, fluoride rinsing appears to reduce the abundance of *S. gordonii* in enamel biofilms while enriching *S. oralis* and *S. parasanguinis*. Although *S. gordonii* is generally associated with oral health and biofilm pH regulation due to its ability to produce ammonium via the arginine-deiminase system [15, 39], it also promotes biofilm maturation by facilitating interspecies coaggregation and the adhesion of secondary colonizers, including periodontal pathogens [40]. Consequently, the biological implications of its reduction remain unclear. In contrast, *S. oralis* and *S. parasanguinis* are commensal early colonizers that contribute to biofilm homeostasis through hydrogen peroxide production and antagonistic activity against cariogenic microorganisms [41, 42]. Additionally, *S. parasanguinis* is arginolytic and contributes to biofilm pH regulation [39], suggesting that their enrichment may be beneficial to biofilm ecology. Beyond these changes in characterized species, fluoride influenced the relative abundance of several uncultivated *Streptococcus* phylotypes, some common to both enamel and dentine and others substrate specific. The ecological significance of these changes will become clearer as these taxa are further characterized.

In both enamel and dentine biofilms, NaF rinsing was associated with lower relative abundance of *Neisseria elongata*, *Prevotella aurantiaca*, and an uncultivated *Bergeyella* species, and higher abundance of *N. mucosa*. The role of *Neisseria* in oral health is complex, as different strains can either produce lactate, therefore decreasing the local pH, or utilize this acid producing lower pKa acids, thus potentially increasing the environmental pH [43]. Likewise, the role of *P. aurantiaca* in oral disease remains unclear, although related species such as *P. intermedia* and *P. nigrescens* are associated with periodontal infections [44]. *Bergeyella* species are commonly associated with health and community stability [45], and their reduced abundance has been linked to high caries experience [46, 47]. A deeper understanding of the role of these species is necessary to define the ecological significance of the observed changes in their abundance within the community.

A relevant aspect of the study design is that all participants used a standardized 1450 ppm fluoride toothpaste during both experimental phases. This approach was chosen to reflect a clinically relevant oral hygiene regimen while maintaining identical baseline conditions across both study phases. Because participants inserted the oral appliance 30 min after toothbrushing, the experimental specimens were exposed after salivary fluoride concentrations had already declined substantially, reaching values below 0.5 ppm [48]. Therefore, the present study evaluated the effect of a 500 ppm NaF rinse compared with deionized water under conditions of standardized fluoride toothpaste use, rather than the isolated effect of a fluoride rinse in a fluoride-free environment. It should be noted, however, that prior exposure to fluoride toothpaste may have reduced the contrast between the experimental conditions and contributed to the relatively subtle microbiome differences observed. Future studies employing a fluoride-free toothpaste during the lead-in and washout phases may help further elucidate the isolated effects of fluoride rinsing on early biofilm composition.

An important limitation of this study is that microbiome sequencing was performed on pooled samples rather than individual participant samples. This approach optimized DNA yield for sequencing; however, extending the biofilm formation period in future studies may generate sufficient biomass without the need for pooling. Consequently, in our study, the analytical unit for the microbiome analyses was the pooled sample, resulting in three biological replicates per treatment/substrate condition. Although the clinical study employed a crossover design, pooling prevented participant-level paired analyses and precluded the assessment of inter-individual variability. Therefore, the statistical advantages of the crossover design could not be fully exploited. To address this limitation, the interpretation of the findings was focused primarily on differences between study groups rather than on individual changes. Furthermore, the limited biological replication requires cautious interpretation of the differential abundance analyses, particularly for low-abundance taxa. Although several taxa were identified as differentially abundant in both enamel and dentine biofilms following fluoride exposure, these findings should be considered exploratory and hypothesis-generating. Confirmation of these observations will require studies analysing individual-level sequencing data.

Although the fluoride rinse induced statistically significant changes in the abundance of several bacterial species in both substrates, many of the altered taxa were present at low relative abundances. Differences involving sparsely represented taxa may reflect random fluctuations, particularly in the context of the pooling strategy used in this study. Moreover, although full-length 16S rRNA gene sequencing provides improved taxonomic resolution compared with short-read approaches, discrimination among some closely related taxa, particularly within the genus Streptococcus, remains challenging. Therefore, the observed species-level changes, especially among closely related streptococci and low-abundant taxa, should be interpreted with appropriate caution. Whether these early subtle alterations affect the subsequent development and maturation of the biofilm remains to be elucidated. Overall, given that microbial interactions and metabolism are highly influenced by environmental factors, including host diet (e.g. sugar intake) and behavioural habits [45], the ecological implications of the changes observed in the present study remain unclear. Investigations integrating metatranscriptomics and metabolic profiling, especially in the presence of disrupting factors such as sucrose exposure, will be essential to clarify the functional roles of the affected taxa and determine whether fluoride promotes changes associated with disease progression or community stability. Curiously, a trend was observed in alpha and beta diversity indexes, which seems to indicate a possible reduction in diversity and a shift in the microbial composition after rinsing with NaF. However, no statistically significant differences were observed, possibly due to the limited sample size used in the study, though this trend warrants further investigation. Furthermore, future studies could evaluate whether other forms of fluoride delivery, such as dentifrices, or other fluoride sources, such as stannous fluoride or sodium monofluorophosphate, also promote similar changes in the relative abundance of specific taxa.

Finally, the exclusive use of sequencing-based analyses represents another limitation of the present study. Although full-length 16 S rRNA gene sequencing provides detailed taxonomic profiling, it cannot characterize the viability or functional properties of detected microorganisms. Recent advances in culturomics have highlighted the complementary value of culture-based approaches for the characterization of previously uncultivated oral microorganisms [49]. Therefore, future studies integrating sequencing with cultivation-based methods may provide a more comprehensive understanding of the effects of fluoride on early oral biofilm formation.

## Conclusions

Within the limitations of this exploratory microbiome analysis, fluoride rinse was associated with subtle differences in the composition of the early biofilms formed on enamel and dentine in the species level, with changes in the relative abundance of less present genera, while the dominant genera and overall diversity were not influenced. Notably, fluoride appears to enrich several abundant taxa associated with oral health, including *S. oralis*, *S. salivarius*, and *S. parasanguinis* in enamel biofilms, and *S. salivarius*, *Rothia aeria*, and an uncultivated *Haemophilus* species in dentine biofilms. These findings raise the hypothesis that fluoride may contribute to oral health not only through its established effects on mineral dynamics but also by favouring health-associated taxa in early biofilms, although these observations should be interpreted cautiously and require confirmation in studies based on individual-level microbiome analyses.

## Supplementary Information

Below is the link to the electronic supplementary material.

Supplementary Material 1

## Data Availability

Datasets generated in the study are available from the corresponding author upon reasonable request.
